# Supplementary material for: Antibiotic Resistance Trends in Recurrent Paediatric Urinary Tract Infections: A Five-Year Single-Centre Experience
Source: Children (Basel). 2025 Nov 18;12(11):1567. doi: 10.3390/children12111567 (PMC12651313; doi:10.3390/children12111567)
Supplement: Supplementary file 1 [file children-12-01567-s001.zip › Figure S1.pdf]

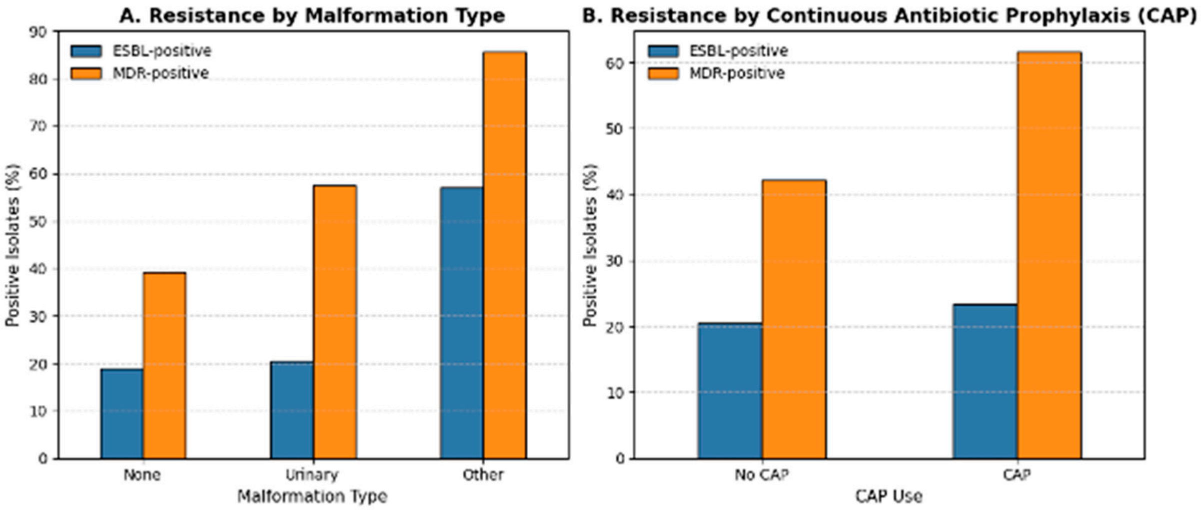

Figure S1. Correlation Between Resistance and Clinical Factors

(A) Prevalence of extended-spectrum β-lactamase (ESBL) and multidrug-resistant (MDR) isolates according to the presence and type of urinary tract malformations. MDR pathogens were significantly more frequent among children with urinary malformations ($p = 0.018$), while no significant association was found between ESBL production and malformation status ($p = 0.061$).

(B) Distribution of ESBL and MDR isolates in relation to continuous antibiotic prophylaxis (CAP) use. Although ESBL production was not influenced by prophylactic treatment ($p = 0.87$), a borderline association was observed between CAP and MDR infections ($p = 0.05$), suggesting that prolonged antibiotic exposure may contribute to the selection of resistant strains. Together, these findings demonstrate that urinary tract malformations and sustained antibiotic pressure represent key contributors to antimicrobial resistance in pediatric recurrent UTIs.
