# Supplementary material for: Antibiotic Resistance Trends in Recurrent Paediatric Urinary Tract Infections: A Five-Year Single-Centre Experience
Source: Children (Basel). 2025 Nov 18;12(11):1567. doi: 10.3390/children12111567 (PMC12651313; doi:10.3390/children12111567)
Supplement: Supplementary file 1 [file children-12-01567-s001.zip › Figure S2.pdf]

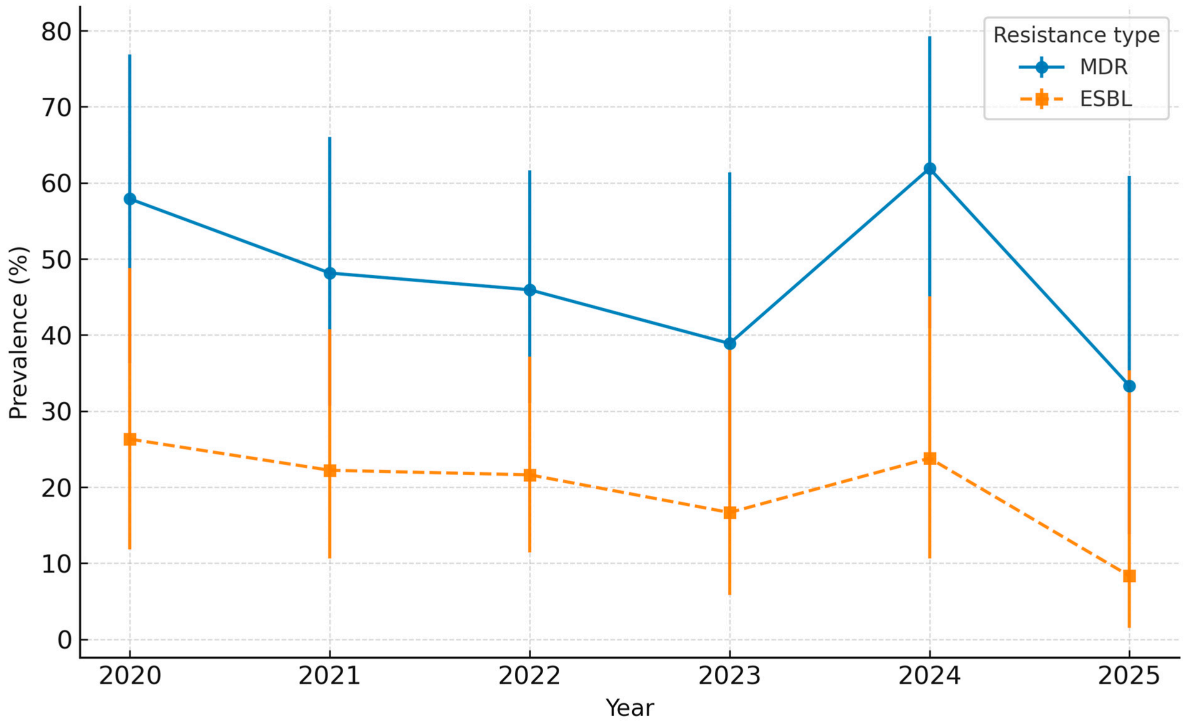

Figure S2. *Temporal evolution of multidrug resistance (MDR) and extended-spectrum β-lactamase (ESBL) positivity among bacterial isolates from recurrent paediatric urinary tract infections (2020–2025).*

MDR prevalence (blue line) and ESBL prevalence (orange line) are shown with Wilson 95% confidence intervals. MDR increased modestly from 41.2% in 2023 to 63.2% in 2024, while ESBL prevalence remained relatively stable (range 17.6–26.3%). No statistically significant linear trend was observed for either MDR (logistic regression OR per year = 0.94, 95% CI 0.75–1.17; p = 0.566; Cochran–Armitage χ^2 = 0.89, p = 0.346) or ESBL.
