# Supplementary material for: Antibiotic Resistance Trends in Recurrent Paediatric Urinary Tract Infections: A Five-Year Single-Centre Experience
Source: Children (Basel). 2025 Nov 18;12(11):1567. doi: 10.3390/children12111567 (PMC12651313; doi:10.3390/children12111567)
Supplement: Supplementary file 1 [file children-12-01567-s001.zip › Table S1.pdf]

Table S1. Antibiotic resistance by class with 95% confidence intervals (n = 134).

Antibiotic class	n	%	95% CI lower	95% CI upper
**Multidrug resistance (MDR ≥3 classes)**	65	48.5	40.2	56.9
**Amoxicillin and derivatives**	31	23.1	16.8	31.0
**No resistance**	20	14.9	9.9	21.9
**Trimethoprim–sulfamethoxazole (TMP/SMX)**	7	5.2	2.6	10.4
**Nitrofurantoin**	6	4.5	2.1	9.4
**Streptomycin/tetracycline**	2	1.5	0.4	5.3
**Cephalosporins**	2	1.5	0.4	5.3
**Gentamicin**	1	0.7	0.1	4.1

Abbreviations: CI, confidence interval; MDR, multidrug resistance (resistance to ≥3 antibiotic classes).
