# Supplementary material for: Antibiotic Resistance Trends in Recurrent Paediatric Urinary Tract Infections: A Five-Year Single-Centre Experience
Source: Children (Basel). 2025 Nov 18;12(11):1567. doi: 10.3390/children12111567 (PMC12651313; doi:10.3390/children12111567)
Supplement: Supplementary file 1 [file children-12-01567-s001.zip › Table S3.pdf]

Table S3. Multivariable logistic regression model for predictors of multidrug resistance (MDR).

Variable	Odds Ratio (OR)	95% CI Lower	95% CI Upper	p-value
**Intercept**	0.614	0.301	1.255	0.181
**malf_any**	2.069	0.763	5.611	0.153
**cap_yes**	1.273	0.448	3.613	0.651
**sex_female**	0.901	0.429	1.896	0.784
**age_years**	1.03	0.947	1.12	0.49

*Model includes all predictors with complete data: malformation (any vs none), continuous antibiotic prophylaxis (CAP), sex, and age (years). Odds ratios (OR) are presented with 95% confidence intervals and p-values. The intercept term is retained for full model transparency. Abbreviations: CI, confidence interval; CAP, continuous antibiotic prophylaxis; OR, odds ratio; MDR, multidrug resistance.
